# Supplementary material for: The Use of Respiratory Effort Improves an ECG-Based Deep Learning Algorithm to Assess Sleep-Disordered Breathing
Source: Diagnostics (Basel). 2023 Jun 23;13(13):2146. doi: 10.3390/diagnostics13132146 (PMC10340311; doi:10.3390/diagnostics13132146)
Supplement: Supplementary file 1 [file diagnostics-13-02146-s001.zip › diagnostics-2400065-supplementary.pdf]

Supplementary Materials:

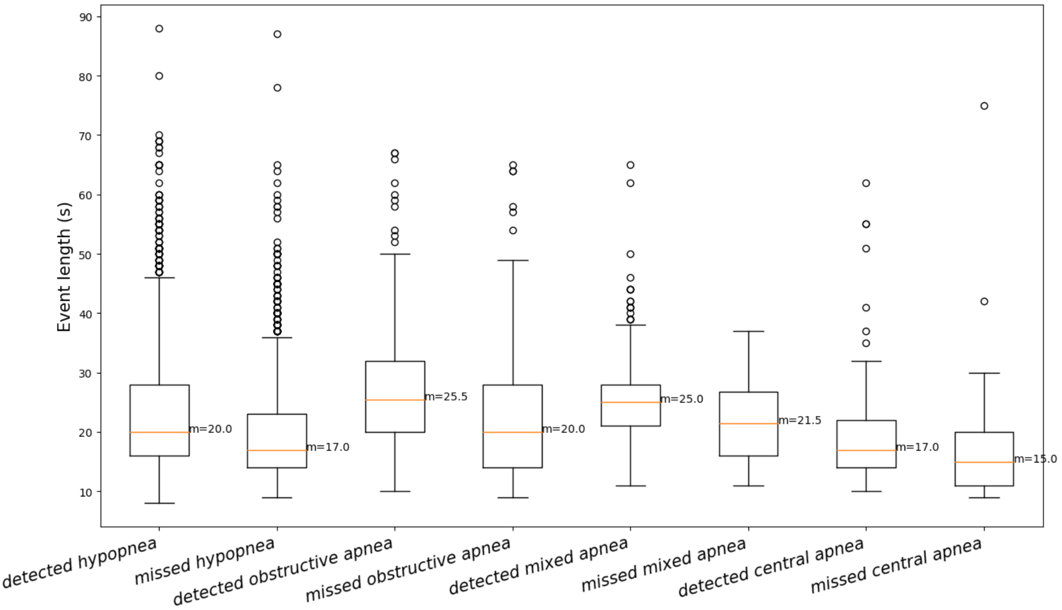

Figure S1. Boxplot illustrating the event length distribution for detected and missed SDB events of all types in the testing set, utilizing RR and EDR as input (m stands for median).

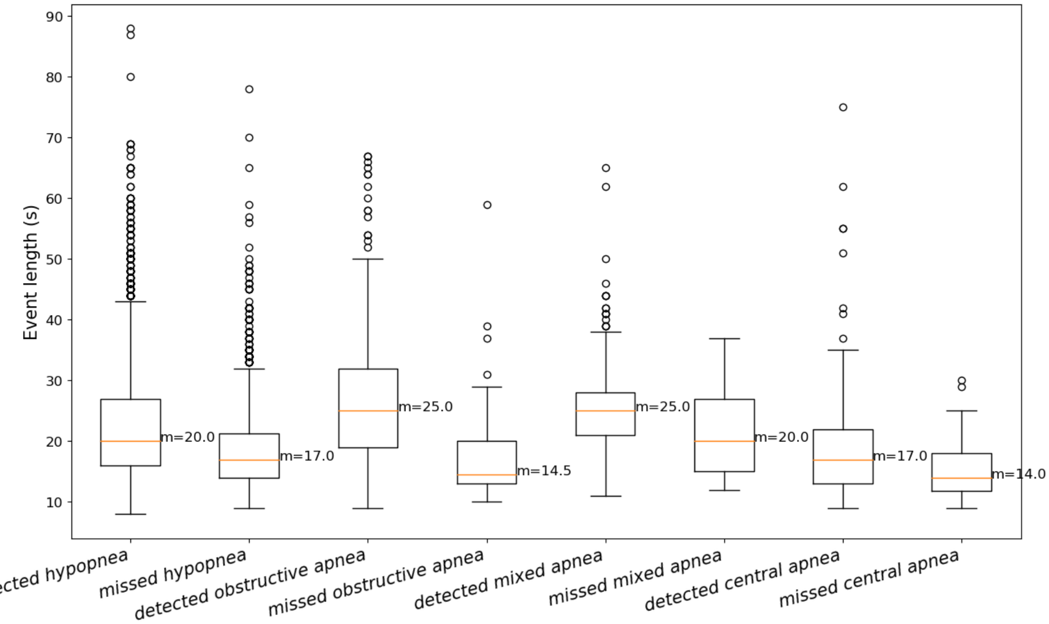

Figure S2. Boxplot illustrating the event length distribution for detected and missed SDB events of all types in the testing set, utilizing RR and RE as input (m stands for median).

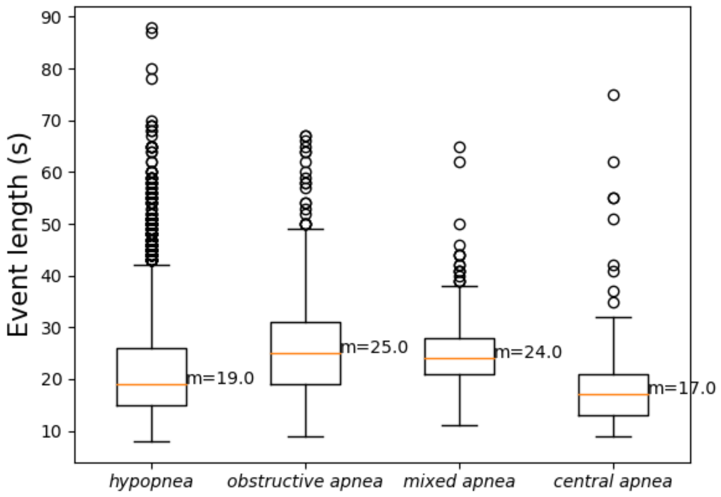

Figure S3. Boxplot illustrating the event length distribution of various types of SDB events in the testing set.

Table S1. Sensitivity of SDB event detection in different sleep stages with RR and RE as inputs

Sleep stage	N1	N2	N3	REM
Number of SDB events	932	2291	125	676
Number of detected SDB events	731	1769	56	512
Sensitivity	0.784	0.772	0.448	0.757

Note: SDB events having overlapped periods with different stages are not included to avoid confusion.

Table S2. Sensitivity of SDB event detection in different body positions with RR and RE as inputs

Body position	right	Supine	Left	Prone
Number of SDB events	1769	2754	1267	142
Number of detected SDB events	1305	2213	989	90
Sensitivity	0.738	0.804	0.781	0.634

Note: SDB events having overlapped periods with different body positions are not included to avoid confusion.
